# Do Adductor Tenotomies Prevent Progressive Migration in Children with Cerebral Palsy? A Systematic Review

**DOI:** 10.2106/JBJS.RVW.24.00093

**Published:** 2024-08-20

**Authors:** Renée Anne van Stralen, Merel Charlotte Rosalie Roelen, Pranai Buddhdev, Max Reijman, Denise Eygendaal, Jaap Johannes Tolk

**Affiliations:** 1 Department of Orthopedics and Sports Medicine, Erasmus MC Sophia Children's Hospital, Rotterdam, the Netherlands; 2 Broomfield Hospital, Chelmsford, United Kingdom

## Abstract

**Background:**

Up to one-third of children with cerebral palsy (CP) develop migration of the hip, and the risk increases with a higher Gross Motor Function Classification System (GMFCS). In progressive hip migration in young children, adductor tenotomy is an accepted treatment option to delay or prevent progressive hip migration. However, there is quite a large variability in reported results. This systematic review aims to determine the effectiveness of a soft-tissue release in the prevention of progressive hip migration in children with CP.

**Methods:**

This systematic review was performed in accordance with the guidelines of the Cochrane Handbook for Systematic Reviews and the Preferred Reporting Items for Systematic Review and Meta-Analysis Protocols statements. Our inclusion criteria were studies describing pediatric, skeletally immature patients with CP and a “hip at risk” of progressive hip migration. Exclusion criteria were simultaneous bony reconstructions, case reports, technical notes, published abstracts, or studies with a follow-up under 1 year postoperatively. The primary outcomes were defined as failure rate (progressive hip migration and/or need for bony surgery, as defined by each paper) and change in migration percentage (MP) at final follow-up. As secondary analyses, we evaluated the outcome after specific subtypes of surgeries and assessed whether performing lengthening of iliopsoas, neurectomy of the anterior branch of the obturator nerve, age at the time of surgery, GMFCS level, and postoperative management impact the outcome.

**Results:**

Our literature search identified 380 titles. Eighty-four articles underwent full-text review, of which 27 met our inclusion/exclusion criteria and were subsequently selected for quantitative analysis. A prevalence meta-analysis was performed including 17 studies (2,213 hips). Mean follow-up ranged from 12 to 148.8 months. The mean preoperative MP was 33.4% (2,740 hips) and 29.9% at follow-up. The overall reported failure rate was 39% (95% confidence interval, 26%-52%). Performing a release of only adductor longus had a failure rate of 87%, whereas more extensive soft-tissue releases showed significantly better results with failure rates ranging from 0 to 44% (p < 0.001). Lengthening of the iliopsoas had no significant impact on failure rate (p = 0.48), nor did performing an obturator neurectomy (p = 0.92).

**Conclusion:**

The failure rate of adductor tenotomies to prevent progressive hip migration appears to be as high as 39% in studies with a varying follow-up. The failure rates are significantly higher when isolated release of the adductor longus is performed. This systematic review supports clinical decision making in children with CP and early hip migration.

**Level of Evidence:**

Level IIA. See Instructions for Authors for a complete description of levels of evidence.

Cerebral palsy (CP) is the most common cause of chronic physical disability of childhood, with a prevalence of 2 to 3 per 1,000 live births. CP is characterized by nonprogressive brain damage occurring before birth or in early infancy and can affect a person's ability to move or maintain balance and posture. Up to one-third of children with CP have migration and subsequent (sub)luxation of the hip with an increasing incidence associated with a higher Gross Motor Function Classification System (GMFCS)^1-5^. Children classified as GMFCS IV/V have reported hip migration rates of 45% to 69.2% and 67% to 89.7%, respectively^2,5,6^.

Hip migration in children with CP has been attributed to several factors. One of these is the spasticity of the hip adductors and flexors leading to the development of contractures^7,8^. Other potential contributing factors related to the muscle imbalance are coxa valga, excessive femoral anteversion, persistent lateral physeal tilt, and inadequate weight bearing^9,10^. The contribution of these factors other than spasticity and contractures is supported by several studies that have shown no difference in the rate of hip dislocations between patients with CP who underwent tone management surgeries and those who did not^11-13^. Progressive hip migration and dislocation can cause pain, disturbed seated balance or standing abilities, difficulty with perineal care, development of decubitus ulcers, and poor quality of life^14-18^.

Surgical treatment of hip migration in individuals with CP, usually limited to hips with a 40% or higher migration percentage (MP), involves hip reconstruction that consists of soft-tissue releases and femoral and/or pelvic osteotomies^19^. These procedures are known to have significant perioperative morbidity, including pain, significant blood loss, and lengthy anesthetic and recovery times and have a relatively high risk of perioperative wound infections^20,21^. Inan et al. described 10% of cases developing a deep or superficial infection^20^, and McNerney et al. described an infection rate of 6.7%^21^. Soft-tissue release (adductor tenotomy) can be considered as an alternative to bony hip reconstruction in early hip migration in children younger than 8 years. Reimers first stated that an adductor contracture could be the primary cause of hip migration in children with CP and should be performed as soon as hip migration is noted^7^. However, the failure rate for adductor-lengthening procedures is relatively high, with 34% to 74% of patients considered as failure due to either progression of migration to >50% or the need for secondary bony surgery^10,22-24^. It has been suggested, however, that adductor surgery has a temporizing effect^10^.

Adductor tenotomies are frequently used as a primary treatment option; however, the available literature has reported varying outcomes. Thus, it is necessary to further clarify the effects of adductor tenotomies and identify the patients who benefit the most. This information will aid in patient selection and facilitate more informed discussions with patients and their families regarding treatment outcomes.

This systematic review aims to determine the effectiveness of a soft-tissue release in the prevention of progressive hip migration in children with CP. Secondary analyses of this systematic review were to determine whether age at surgery, surgical technique, preoperative hip migration, and duration of follow-up affect the failure rate at follow-up.

## Methods

This systematic review was performed in accordance with the guidelines of the Cochrane Handbook for Systematic Reviews and the Preferred Reporting Items for Systematic Review and Meta-Analysis Protocols (PRISMA-P) statements^25^. The protocol followed was registered with and accepted by the International Register of Systematic Reviews on September 7, 2022 (CRD42022354917).

### Information Sources and Search Terms

A thorough search of the literature was performed through PubMed, MEDLINE, Cochrane, and EMBASE to identify original articles qualified as level IV or higher. Appropriate search terms, including Boolean operators suitable for each database, were applied for each database (Appendix 1). Cross-reference search results of the included studies and gray literature were included when available. The literature search was performed in April 2024. Studies published in other languages than English were excluded.

Our inclusion criteria were pediatric (age younger than 18 years), skeletally immature patients with CP undergoing soft-tissue surgery for treatment of progressive hip migration. Exclusion criteria included previous proximal femur or pelvic operations, case reports, technical notes, and published abstracts or reports with a follow-up less than 1 year postoperatively.

**Fig. 1:**
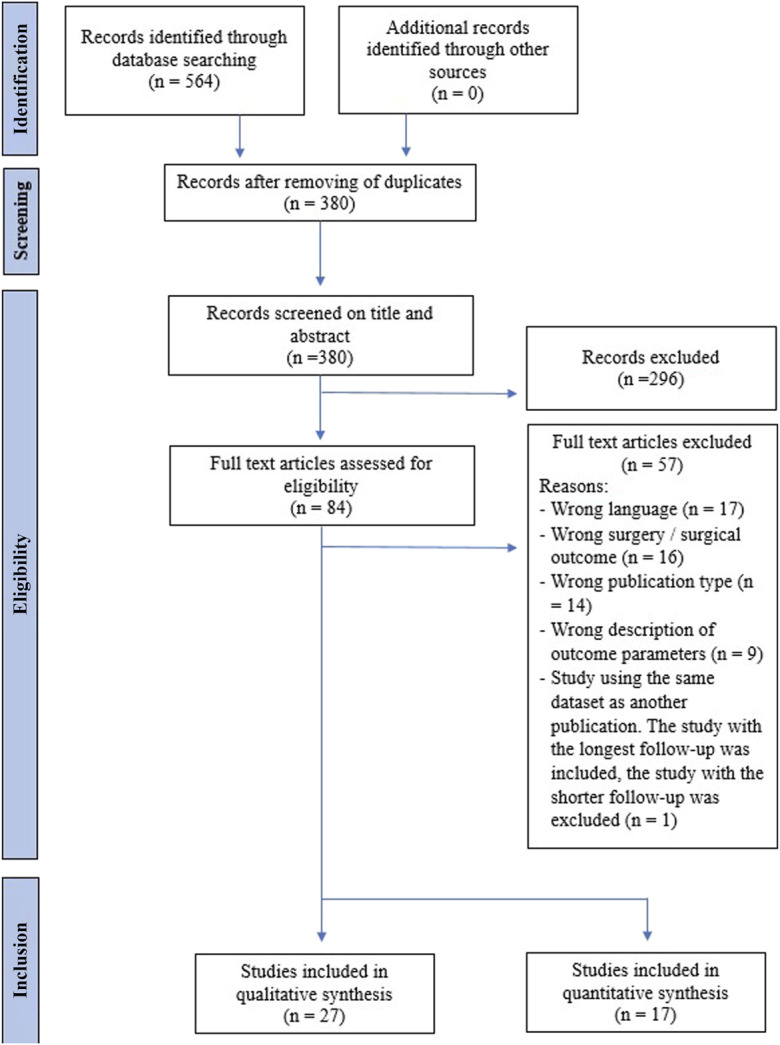
Flowchart of inclusion.

The PRISMA flowchart is illustrated in Figure 1. Two independent reviewers (R.A.v.S. and P.B.) separately and blinded to each other's results conducted the screening of search results against the inclusion/exclusion criteria based on title, abstract, and keywords. Disagreements were resolved by an independent third author (J.J.T.).

### Assessment of Risk of Bias

Risk of bias was assessed for all studies, using the ROBIN-I checklist^26^ as recommended in the Cochrane Handbook for Systematic Reviews^27^.

### Statistical Analysis

The primary outcome was failure rate after at least 1 year of follow-up. Failure is defined by every study specifically, but in general, it is defined as the need for bony surgery or progression of MP. Different studies use slightly different MPs as a threshold for the definition of failure. The studies included in the systematic review did not define developing a windswept deformity or adduction contracture as a failure outcome. The study by Abel et al. is the only study that evaluated windswept deformity.

Prevalence meta-analysis was performed by using Stata (Stata Statistical Software: Release 18; StataCorp LLC, 2019). Statistical significance was set at the 5% level.

Subgroup analyses were conducted to investigate the impact of age at the time of surgery on the failure rate, as well as the effects of individual or combined adductor tenotomies, performing lengthening of the iliopsoas, performing neurectomy of the anterior branch of the obturator nerve, and postoperative abduction splinting on the outcome. These analyses were performed by a prevalence meta-analysis with a subgroup analysis. In addition, we examined whether preoperative MP at the time of surgery (<40% or >40%) had an impact on failure rate at follow-up. To assess the potential impact of the duration of the available follow-up, subgroups were created with a mean follow-up of 0 to 24, 24 to 48, 48 to 72, 72 to 96 months, and more than 96 months.

MPs were calculated by creating a weighted mean divided by the cumulative number of patients described. This was performed for the total population and for the studies describing MP in both ambulant and nonambulant children separately.

Secondary outcomes were MP at follow-up and complication rates, as graded by the Clavien-Dindo System.

## Results

Our literature search identified 266 titles. Eighty-one articles underwent full-text review, of which 25 met our inclusion/exclusion criteria and were subsequently selected for quantitative analysis (Fig. 1).

The study characteristics and outcomes for all included studies are summarized in Table I. The mean age at surgery ranges from 3.4 years to 8.5 years, and the mean follow-up ranges from 12 to 148.8 months.

**TABLE I:** Study Characteristics and Reported Outcomes of Patients with CP Undergoing Adductor Tenotomies*†

A: Study Characteristics						
Reference	Study Design	Time Frame for Inclusion	No. of Hips (Patients)	GMFCS Classification No. of Hips (Patients)	Age, yr (Range)	FU, mo, Mean (Range)
Abel et al., 1999^28^	Retrospective case series	1988-1993	30 (15)	Unknown	4.6	47
Asfuroglu et al., 2022^29^	Retrospective case series	2002-2012	58 (30)	GMFCS II: 5 (3) GMFCS III: 15 (8) GMFCS IV: 38 (19)	8.3 (3-18)	29.6
Asma et al., 2022^11^	Retrospective comparative study	Unknown	93 (48)	GMFCS IV: 9 patients GMFCS V: 39 patients	4.3 (1.9-9.4)	110.3
Bishay, 2008^30^	Prospective cohort	September 2003-September 2004	100 (50)	Nonambulant 72 (36) Ambulant 28 (14)	3.6 (3-5)	27.6 (24-36)
Bozinovski et al., 2008^31^	Unknown	Unknown	11 (11)	Nonambulant 11 (11)	8.5 (5.9-11.7)	48
Bozinovski et al., 2008^32^	Unknown	Unknown	44 (22)	Unknown	5.4 (2.2-7.8)	12
Cobeljic et al., 1994^33^	Unknown	Unknown	20 hips only adductor tenotomy 42 (25) hip release	Unknown	4.5 7 (3-13)	78 60
Cottalorda et al., 1998^34^	Retrospective case series	1977-1995	57 (30)	Nonambulant: 27 patients Ambulant: 3 patients	6.1 (2.5-13)	75 (24-240)
Guglielmetti et al., 2010^35^	Retrospective case series	1991-2006	88 (44)	Nonambulant: 29 patients Ambulant: 15 patients	6.4 (0-20)	70.8
Kalen and Bleck, 1985^22^	Retrospective case series	1962-1982	97 (56)	Group I (adductor): Nonambulant: 27 hips Ambulant: 12 hips Group II (adductor-psoas): Nonambulant: 27 hips Ambulant: 31 hips	4.9 (1.5-17)	53.6 (18-154)
Khot et al., 2008^36^	Prospective cohort	2002-2005	32 (16)	GMFCS III: 10 (5) GMFCS IV: 22 (11)	Range 2-6	24
Kiapekos et al., 2019^37^	Registry based retrospective cohort	1995-2012	258 (129)	GMFCS I-II-III: 26 (13) GMFCS IV: 74 (37) GMFCS V: 158 (79)	4.9 (SD 2.2)	96
Onimus et al., 1991^38^	Unknown	Unknown	40 (24)	Nonambulant: 40 (24)	4.0	36 (12-84)
Pap et al., 2005^39^	Retrospective case series	1980-1999	76 (41)	Unknown	4.9 (2-12)	36
Presedo et al., 2005^40^	Retrospective case series	March 1988-January 1991	129 (65)	Nonambulant: 47 patients Ambulant: 18 patients	4.4 (1.9-8)	129.6
Reimers and Poulsen, 1984^41^	Unknown		29 (19)	Unknown	5.9 (2.1-12.3)	20 (7-33)
Schulz et al., 1984^42^	Retrospective case series	1960-1980	22	Unknown	5.0 (2-12)	63.6 (12-192)
Shaheen, 1991^43^	Unknown	April 1985-April 1989	71 (36)	Nonambulant: 36 patients	4.5 (2-10)	24 (12-36)
Shore et al., 2012^10^	Retrospective case series	January 1994-December 2004	660 (330)	GMFCS II: 33 patients GMFCS III: 55 patients GMFCS IV: 103 patients GMFCS V: 139 patients	4.2	85 (24-161)
Silver et al., 1985^44^	Retrospective case series	Unknown	76 (39)	Nonambulant: 39 patients	3.9	45 (12-92)
Spruit and Fabry, 1997^45^	Retrospective case series	1986-1994	17 (12)	Unknown	6.05 (2-13.5)	48.6 (30-96)
Terjesen, 2017^46^	Retrospective case series	2006-2011	72 (37)	GMFCS III: 9 patients GMFCS IV: 10 patients GMFCS V: 18 patients	5.0 (2.8-7.2)	87.6 (61.2-117.6)
Terjesen et al., 2005^47^	Retrospective case series	1986-1991	78 patients	Ambulant: 35 patients Nonambulant: 43 patients	8 (2-17)	120 (19.2-192)
Turker and Lee, 2000^23^	Retrospective case series	Unknown	90 (45)	Unknown	4.9	97.2
Wagner and Hägglund, 2022^24^	Retrospective case series	Patients born in 2000-2011, surgery before age 12 yr	158 (158)	GMFCS III: 16 patients GMFCS IV: 57 patients GMFCS V: 85 patients	5.3 (1.5-12)	30
Wong et al., 2022^48^	Retrospective case series	1998-2015	64 (33)	GMFCS I-III: 20 GMFCS IV-V: 13	5.9 (SD 2.7)	148.8 (60-216)
Yang et al., 2008^49^	Retrospective case series	February 2004-March 2007	120 (60)	GMFCS I: 2 patients GMFCS II: 12 patients GMFCS III: 21 patients GMFCS IV: 18 patients GMFCS V: 7 patients	3.4 (2.2-5.7)	22.5 (19-52)

B: Reported Outcomes					
Reference	Postoperative Management	Surgical Procedure	FU, mo (Range)	Outcome	Rate of Failure
Abel et al., 1999^28^	Not described	Adductor tenotomy, usually combined with psoas tenotomy	47	Mean MP preop 33.5% Mean MP at FU 34.0%	Failure rate^a^: 74%
Asfuroglu et al., 2022^29^	Abduction cast for 3 weeks Night-time abduction orthosis for 3 months	Unknown	29.6	Mean MP preop 28.2% (±10.1%) Mean MP at FU 22.2% (±14.6%)	Not described
Asma et al., 2022^11^	Physical therapy	Adductor longus, gracilis, iliopsoas ± adductor brevis, simultaneous hamstring release if indicated	110.3	Not described	Failure rate^d^: 65%
Bishay, 2008^30^	Abduction cast for 3 weeks	Adductor longus, gracilis, rectus, and iliopsoas tenotomy	27.6 (24-36)	Not described	Failure rate: 0%
Bozinovski et al., 2008^31^	Abduction cast for 7 days	Adductor longus, gracilis, brevis, rectus, sartorius, and iliopsoas tenotomy	48	Mean MP preop 100% Mean MP at FU 39.5% (±6.8%)	Not described
Bozinovski et al., 2008^32^	Abduction cast for 7 days	Adductor longus, gracilis ± brevis ± psoas	12	Mean MP preop 56.0% Mean MP at FU 43.8%	Not described
Cobeljic et al., 1994^33^	Unknown	Adductor tenotomies alone Adductor longus, brevis, rectus, psoas, iliac crest, and knee flexors	78 60	Mean MP preop 45% Mean MP at FU 25%	Failure rate^a^: 100% Failure rate^a^: 3%
Cottalorda et al., 1998^34^	Abduction cast 3 weeks, followed by night splinting for 3 months	Adductor longus, gracilis, neurectomy in 23 cases	75 (24-240)	Not described	Failure rate^b^: 35.1%
Guglielmetti et al., 2010^35^	Abduction cast 3 weeks	Adductor longus, adductor brevis and gracilis	70.8	Not described	Failure rate^b^: 48%
Kalen and Bleck, 1985^22^	Unknown	Adductor longus ± psoas tenotomy	53.6 (18-154)	Not described	Failure rate^b^: 37.7%
Khot et al., 2008^36^	Abduction cast 3 weeks	Adductor longus and gracilis and chemodenervation of the anterior branch of the obturator nerve	24	Mean preop MP 29.0% Mean postop MP 20.9%	Failure rate^a^: 9.4%
Kiapekos et al., 2019^37^	Unknown	Adductor tenotomy ± psoas tenotomy	96	Mean preop MP 47% (±12%)	Failure rate^c^: 48%
Onimus et al., 1991^38^	Abduction cast 2-3 weeks	Adductor longus, gracilis, psoas, and neurectomy	36 (12-84)	Mean preop MP 46.8% Mean MP at FU 41.3%	Failure rate^a^: 35%
Pap et al., 2005^39^	Abduction cast 3-4 weeks	Adductor longus	36	Nonambulant: Mean preop MP 35.99% (±15.58%) Mean MP ay FU 38.9% (±21.39) Ambulant: Mean preop MP 31.09% (±13.77%) Mean MP at FU 22.15% (±13.5%)	Not described
Presedo et al., 2005^40^	Functional	Adductor longus, gracilis, adductor brevis, neurectomy if migration >40% in nonambulant, psoas in nonambulant, proximal hamstrings	129.6	Mean preop MP 34% (range 5%-80%) Mean MP at FU 18% (range 0%-100%)	Failure rate^f^: 30%
Reimers and Poulsen, 1984^41^	Abduction cast 4 weeks	Adductor tenotomy, 14 cases neurectomy, 3 cases with psoas tenotomy	20 (7-33)	Mean preop MP 32% (range 9%-40%) Mean MP at FU 29% (range 8%-54%)	Not described
Schulz et al., 1984^42^	Unknown	Adductor tenotomy with neurectomy	63.6 (12-192)	Mean preop MP 28% (±4%) Mean MP at FU 23% (±5%)	Not described
Shaheen, 1991^43^	Abduction cast, duration not described	Adductor longus, gracilis and neurectomy	24 (12-36)	Mean preop MP 40% (range 30%-90%)	Not described
Shore et al., 2012^10^	Abduction cast 3 weeks	Adductor longus and gracilis ± brevis ± psoas ± neurectomy	85 (24-161)	Mean preop MP 43% (range 21%-64%)	Failure rate^c^: 68%
Silver et al., 1985^44^	Abduction cast 3 weeks	Adductor longus, brevis and gracilis ± psoas	45 (12-92)	Mean preop MP 43.7% Mean MP at FU 34.6%	Failure rate^a^: 19.7%
Spruit and Fabry, 1997^45^	Unknown	Adductor longus and psoas	48.6 (30-96)	Mean preop MP 36.8% (±22.0%) Mean MP at FU 35.2% (±21.7%)	Not described
Terjesen, 2017^46^	Abduction cast 5-6 weeks	Adductor longus and gracilis ± partial myotomy of adductor brevis	87.6 (61.2-117.6)	Worst hip: Mean preop MP 47% Mean postop MP 37%	Failure rate^d^: 29.7%
Terjesen et al., 2005^47^	Abduction cast 5-6 weeks	Adductor longus and gracilis ± partial myotomy of adductor brevis ± psoas	120 (19.2-192)	Mean preop MP 38%	Failure rate^e^: 17.9% + Failure rate^a^: 14.1%
Turker and Lee, 2000^23^	Unknown	Adductor longus, brevis, gracilis ± psoas	97.2	Mean preop MP 38.9% Mean MP at FU 31.4%	Failure rate^a^: 58%
Wagner and Hägglund, 2022^24^	Unknown	Unknown	30		Failure rate^a^: 34.2%
Wong et al., 2022^48^	Abduction cast 6 weeks or flexion abduction brace	Adductor longus, gracilis, iliopsoas or half of adductor brevis	148.8 (60-216)	Mean preop MP 36.1%	Failure rate^a^: 12.1%
Yang et al., 2008^49^	Unknown	Unknown	22.5 (19-52)	Mean preop MP 35.37% (±10.57%) Mean MP at FU 31.8% (±11.9%)	Not described

* CP = cerebral palsy, GMFCS = Gross Motor Function Classification System, FU = follow-up, MP = migration percentage, postop = postoperative, and preop = preoperative.

† Definitions of failure as described in the original study: ^a^Progressive displacement; additional surgery required; revision soft-tissue surgery, varus osteotomy, or pelvic osteotomy. ^b^“Bad” result defined as an increase in MP >10%. ^c^Underwent osseus reconstruction; underwent revision soft-tissue surgery; MP >50%, not yet treated; MP 100%, underwent salvage surgery. ^d^MP ≥50% at FU, underwent secondary surgery. ^e^MP ≥80% at FU, underwent secondary surgery. ^f^MP ≥ 60% at FU.

### Failure Rate

**Fig. 2:**
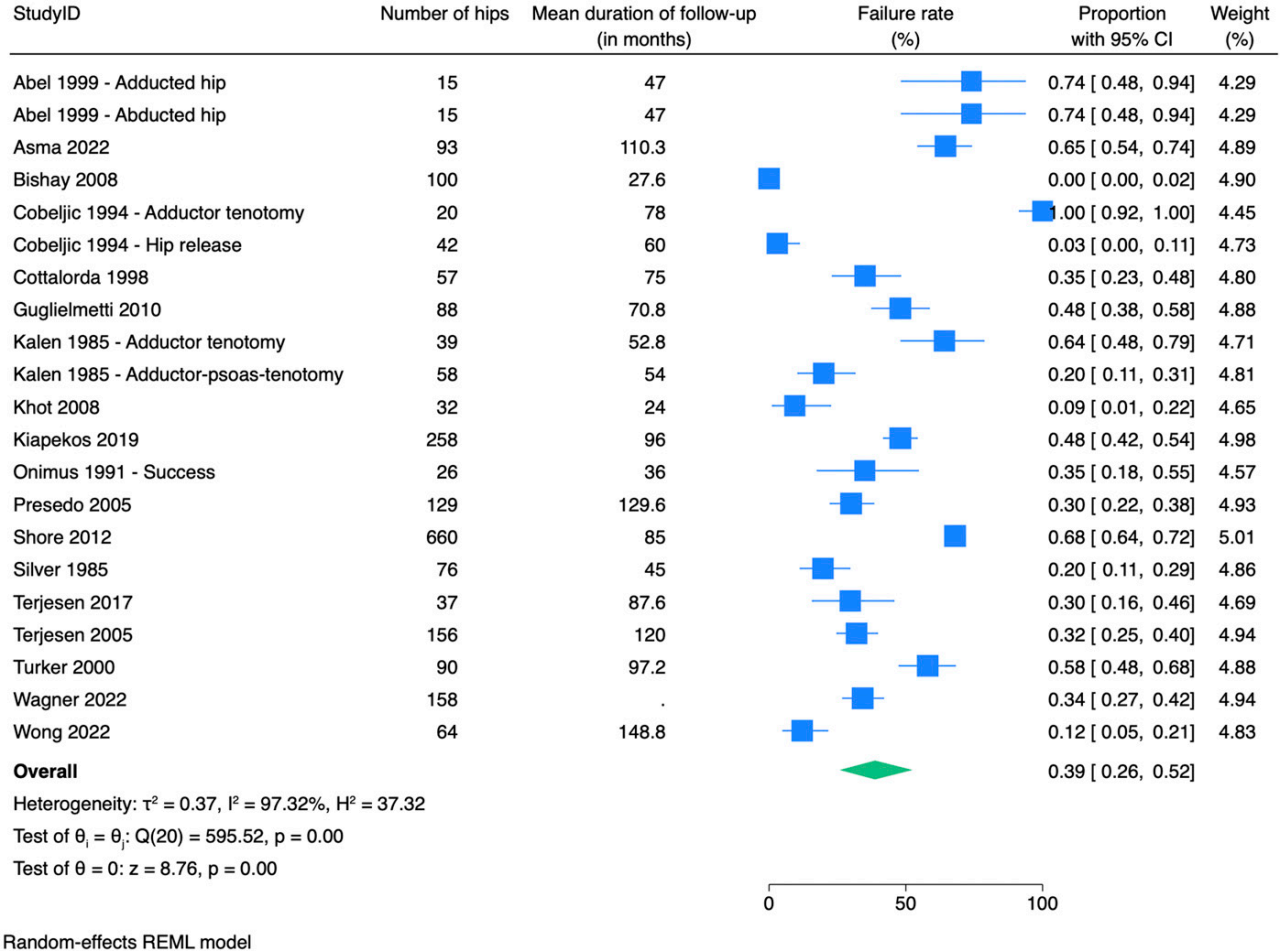
Meta-analysis of failure rate after adductor tenotomies. CI = confidence interval.

A prevalence meta-analysis on the 17 studies that mentioned failure rate (total of 2,070 hips) showed an overall failure rate of 39% (95% confidence interval, 26%-52%) at follow-up. The follow-up ranged from 24 to 148.8 months (Fig. 2).

Failure rates were defined as progressive hip migration (>10%), additional surgery, or a MP higher than a certain percentage at follow-up. Developing a windswept deformity was not included as a failure by the included studies. Abel et al. assessed the pelvic femoral angle as a measurement for windswept deformity and reported no impact of soft-tissue release on windswept deformity^28^.

**Fig. 3:**
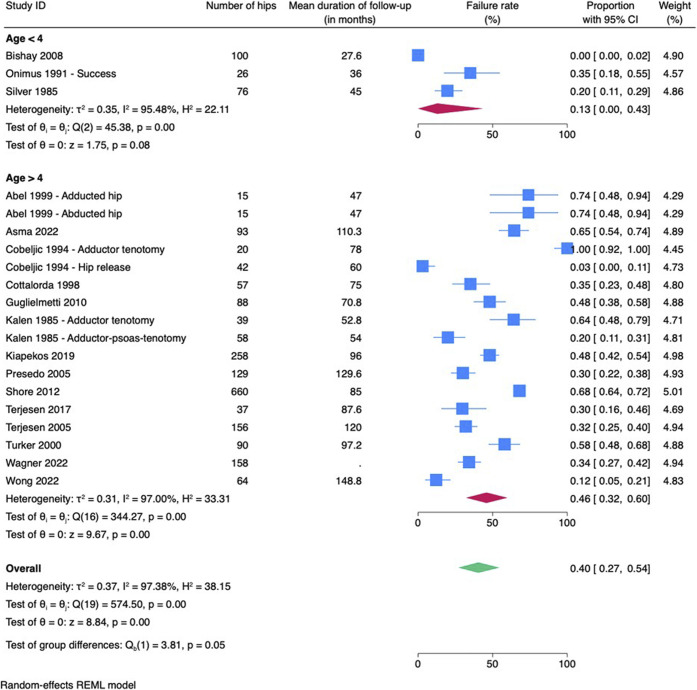
Meta-analysis of patients undergoing adductor tenotomies age younger than 4 or older than 4 years. CI = confidence interval.

**Fig. 4:**
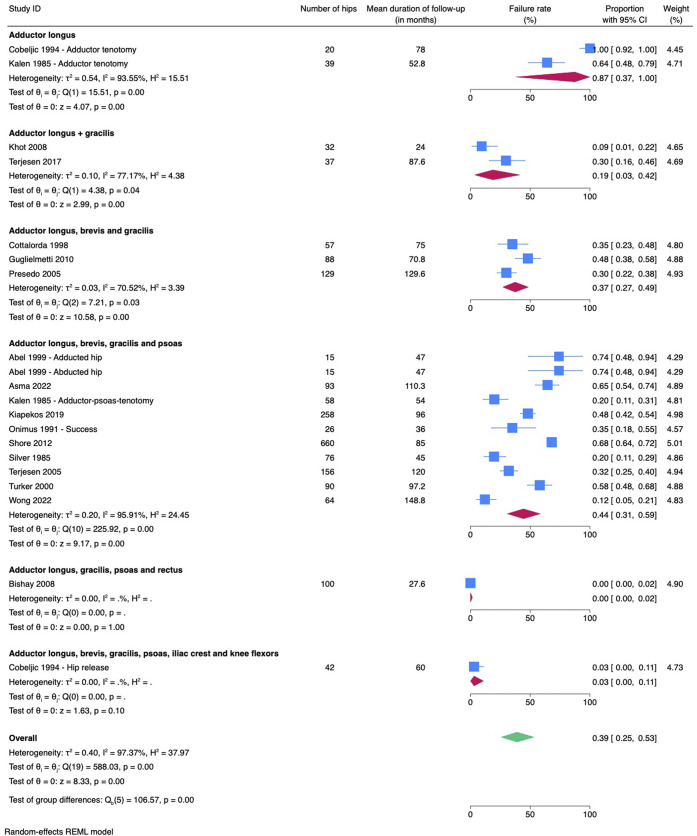
Meta-analysis comparing different types of surgeries. CI = confidence interval.

Failure rates were higher in the group of children who underwent surgery after the age of 4 years (46%) compared with the group that underwent surgery before the age of 4 years (13%). However, this difference was not significant (p = 0.05) (Fig. 3). Performing a release of only adductor longus had a failure rate of 87%, whereas more extensive soft-tissue releases showed significantly better results with failure rates ranging from 0 to 44% (p < 0.001) (Fig. 4). Lengthening iliopsoas had no significant impact on failure rate (p = 0.48). Performing an obturator neurectomy had no significant impact on the failure rate (p = 0.81). Comparing different modalities of postoperative management showed a failure rate of 47% in the group of patients with functional postoperative management and a failure rate of 19% in the group that underwent abduction casts or orthoses postoperatively (p = 0.16).

Nine studies described the failure rate specifically for GMFCS IV-V (nonambulant) and GMFCS I-III (ambulant) children. Combining the data from these studies, the overall failure rate was 19% in the ambulant population (n = 289) and 41% in the nonambulant population (n = 886). This difference was not significant (p = 0.14).

Dividing the available studies into subgroups based on the mean follow-up available, there were no significant differences in failure rates (p = 0.55). Comparing the studies describing a mean preoperative MP of <40% with the studies describing a mean preoperative MP of >40%, there was no significant difference in failure rate at follow-up (p = 0.49). Notably, 1 study described a failure rate after adductor tenotomies in a subgroup of 54 hips of a total of 158 patients with a mean preoperative MP >50%. Their cohort describing GMFCS III-V children showed a reoperation rate of 34.2% at 30-month follow-up^24^. In 51 children, femoral osteotomy was performed, and in 3 cases, revision soft-tissue release was performed. The indication for additional surgery was based on the treating surgeon's discretion and was considered when the MP remained above 40%^24^.

### Migration Percentage

Looking at MP, the mean preoperative MP was 33.4% (2,740 hips) and 29.9% at follow-up. In the studies describing adductor tenotomies in GMFCS I-III children, the mean preoperative MP was 34.9% (266 hips) and 23.2% at follow-up. In the studies describing GMFCS IV-V children, the mean preoperative MP was 43.8% (800 hips) and 34.0% at follow-up.

### Complication Rates

Six studies described complication rates in their cohort. These studies described a total of 688 patients (1,218 hips), and the overall weighted mean complication rate was 2.1%.

Bishay^30^, Kiapekos et al.^37^, and Presedo et al.^40^ described no postoperative complications. Shaheen described a complication rate of 6% and that all these were minor complications^43^. Shore et al. described that 6 of 330 patients experienced a complication: 4 patients developed heel ulcers, 1 patient had a wound infection, and 1 patient experienced prolonged postoperative pain^10^. Terjesen described minor complications in 6 patients in their cohort of 78 patients^47^.

### Risk of Bias

Findings are summarized in Table II and show the overall risk for bias as moderate. Most studies are retrospective and do not report on information regarding potential missing data. Next to that, most studies do not describe results separated for each GMFCS classification.

**TABLE II:** Risk of Bias Assessment

Reference	Bias due to Confounding	Bias in Selection of Participants	Bias in Classification of Intervention	Bias due to Deviations from Intended Intervention	Bias due to Missing Data	Bias in Measurement of Outcomes	Bias in Selection of the Reported Results	Overall Risk of Bias
Abel et al., 1999^28^	Moderate	Moderate	No information	Low	Low	Low	Moderate	Moderate
Asfuroglu et al., 2022^29^	Moderate	Moderate	Low	Low	Low	Low	Low	Moderate
Asma et al., 2022^11^	Low	Low	Low	Low	Low	Moderate	Low	Moderate
Bishay, 2008^30^	Moderate	Low	Low	Low	No information	Low	Moderate	Moderate
Bozinovski et al., 2008^31^	Moderate	Low	Low	Low	Low	Low	Low	Moderate
Bozinovski et al., 2008^32^	Moderate	Moderate	Low	Low	No information	Low	Moderate	Moderate
Cobeljic et al., 1994^33^	Moderate	Moderate	Low	Low	No information	Low	Moderate	Moderate
Cottalorda et al., 1998^34^	Moderate	Low	Low	Low	No information	Low	Moderate	Moderate
Guglielmetti et al., 2010^35^	Moderate	Low	Low	Low	No information	Low	Moderate	Moderate
Kalen and Bleck, 1985^22^	Low	Low	Low	Low	Low	Low	Low	Low
Khot et al., 2008^36^	Moderate	Low	Low	Low	No information	Low	Low	Moderate
Kiapekos et al., 2019^37^	Low	Low	Low	Low	Low	Low	Low	Low
Onimus et al., 1991^38^	Low	Moderate	Low	Low	No information	Low	Low	Moderate
Pap et al., 2005^39^	Moderate	Low	Low	Low	No information	Low	Low	Moderate
Presedo et al., 2005^40^	Moderate	Low	Low	Low	No information	Low	Low	Moderate
Reimers and Poulsen, 1984^41^	Moderate	Serious	Moderate	Low	No information	Low	Moderate	Serious
Schulz et al., 1984^42^	Moderate	Low	Low	Low	No information	Low	Moderate	Moderate
Shaheen, 1991^43^	Moderate	Low	Low	Low	No information	Low	Moderate	Moderate
Shore et al., 2012^10^	Low	Low	Low	Low	Low	Low	Low	Low
Silver et al., 1985^44^	Moderate	Low	Low	Low	No information	Low	Moderate	Moderate
Spruit and Fabry, 1997^45^	Moderate	Low	Low	Low	No information	Low	Moderate	Moderate
Terjesen, 2017^46^	Low	Low	Low	Low	No information	Low	Moderate	Moderate
Terjesen et al., 2005^47^	Moderate	Low	Low	Low	Low	Low	Moderate	Moderate
Turker and Lee, 2000^23^	Moderate	Moderate	Low	Low	No information	Low	Moderate	Moderate
Wagner and Hägglund, 2022^24^	Low	Low	Low	Low	Low	Low	Low	Low
Wong et al., 2022^48^	Moderate	Moderate	Low	Low	Low	Low	Low	Moderate
Yang et al., 2008^49^	Low	Low	Low	Low	No information	Low	Moderate	Moderate

## Discussion

This systematic review and meta-analysis pools data from 27 studies reporting on 2,740 hips. The results on primary outcome show a significantly higher failure rate when only adductor longus is released. Lengthening the iliopsoas and performing a neurectomy of the anterior branch of the obturator nerve did not impact the failure rate. Additionally, a higher preoperative MP and a longer available follow-up period did not influence the failure rate. There is a trend toward a lower failure rate when adductor tenotomies are performed before the age of 4 years, but this difference is not significant.

Not all studies present treatment results in the same way. Most common options are either by presenting failure rate or as MP at follow-up. Our analysis shows that a discrepancy exists between these 2 methods of presenting treatment results with the analyses looking at MPs showing a relatively better outcome compared with the studies describing failure rate as an outcome. It is plausible that the patients undergoing bony reconstruction are excluded from the analysis of MP at follow-up because they can no longer be measured to reflect the results of the soft-tissue release. Because it is not always described whether all patients are included in the final analysis of MP, this could have led to potential bias. This is the reason why failure rate should be the outcome parameter of interest in this type of study.

Most studies included in this systematic review describe outcome in terms of a failure rate (17/27 reporting on 2,213 hips). Failure in these studies is defined as progressive hip migration and/or the need for additional bony surgery. It can be argued that the goal for adductor tenotomy is not to prevent bony surgery in all cases but potentially postpone bony surgery or delay progressive hip migration^10^. If an adductor tenotomy is performed in an early stage of hip migration in a young child and additional bony surgery can be postponed for several years, bony reconstruction can be performed in an older patient. There are 2 advantages to operating at a later age. The first being that the surgery is technically easier as the patient is bigger. The other advantage is that the risk of revision surgery due to recurrent valgus after the bony reconstruction is smaller when a femoral osteotomy is performed at a later age^50^. The term “failure rate” should, therefore, be interpreted in the right perspective.

When considering the impact of the type of surgery on failure rate, our hypothesis is that only lengthening adductor longus has a smaller effect on range of motion and ultimately hip morphology. Performing a lengthening of iliopsoas had no significant impact on failure rates. Some studies did not provide information on whether the surgeon aimed for a specified gain in range of motion or abduction threshold at the end of the procedure. Studies did not routinely report on a comparison of postoperative with preoperative abduction range, nor did they report on postoperative adductor spasticity. As this information is lacking in most studies, it is challenging to draw definitive conclusions on if and how the extent of soft-tissue releases was tailored according to individual patients. It might well be that the abduction reached at the end of the procedure is more important than the surgical tendon–lengthening details.

Soo et al. found a very clear relationship between GMFCS classification and the incidence of progressive hip migration^5^. In addition, Shore et al. concluded that GMFCS score was a strong predictor for the failure rate and need for subsequent bony surgery in a large, retrospective cohort study^10^. Combining the data from several studies in this meta-analysis, there appears to be some difference in failure rate, but the relationship is not statistically significant. A possible explanation for not achieving statistical significance could be the wide distribution of results within and across the included studies. This may be partially attributed to confounding factors that could not be controlled for in the analysis. Ambulant children included in the studies used for this meta-analysis may have exhibited relatively poorer scores on other important prognostic factors for adductor tenotomy treatment failure, compared with nonambulant children. This is supported by the fact that these children developed hip migration, despite having relatively good functional abilities.

Traditionally, the cause of progressive hip migration was thought to be related to spasticity and subsequent contractures leading to hip migration^51,52^. Ulusaloglu et al. suggested that similar features in hypertonic and hypotonic conditions, such as abductor muscle weakness and lack of weight bearing, may be more important factors contributing to proximal femoral lateral physeal tilt^52^. This is supported by the results found by Asma et al., who described no differences in rate of progressive hip migration despite tone management with intrathecal baclofen^11^. Next to that, treatment of spasticity with intramuscular botulinum toxin A combined with abduction hip bracing has been explored as an alternative treatment approach; however, the available evidence suggests this combination provides inadequate benefits to be recommended as the primary treatment method^29,53^.

Ulusaloglu et al. proposed that early strategies aimed at addressing proximal femoral physeal growth may be more effective to treat early hip migration than only addressing spasticity or contractures^52^.

Especially in more severely affected children over 4 years of age, the failure rate is relatively high after adductor tenotomies. For this group of patients, guided growth of the proximal femur has been described as a successful strategy for treating coxa valga and reducing the rate of hip migration in children with CP^54-60^. A recent meta-analysis on results of this technique showed a decrease in mean MP and acetabular index^57^. The overall complication rate was low, but up to 43% of children required screw revision due to growing off the screws. Overall, proximal femur-guided growth is a promising treatment option in children with early-stage hip migration, especially in those older than 4 years.

There are several strengths to this systematic review. First of all, there was a clear search strategy, and a very large number of studies and patients could be included in the analysis. In addition, it focuses not only on primary outcomes but also includes a thorough analysis of several subgroups and potential confounding factors.

Some limitations need to be addressed. There is a wide variety in available follow-up in the included studies. Differences in follow-up could affect the failure rates described. With this type of surgery, a follow-up less than 24 months is relatively short, as surgeons are likely to await the results of their surgery before considering additional bony surgery. Only 2 studies with a follow-up less than 24 months were included in this systematic review^32,41^.

However, a subgroup analysis looking at follow-up showed no differences in failure rates. Next to that, one of the limitations of this review is that not all mean MPs and standard deviations were available for analysis and that not all studies can be included in both the analysis looking at failure rates and the analysis of MPs. In addition, not all studies describe their patient population in terms of GMFCS classification, which makes it more difficult to interpret their results.

In conclusion, the failure rate of adductor tenotomies to prevent progressive hip migration appears to be as high as 39% in studies with a follow-up ranging from 1 to 10 years. The results are slightly better in children younger than 4 years and children who are less severely affected and may, therefore, be used as a temporizing measure in early hip migration to postpone bony hip surgery. Further research should focus on minimal invasive, early interventions to improve outcome and decrease treatment burden for this vulnerable patient group.

### Source of Funding

No funding was received for this study.

## Appendix

Supporting material provided by the authors is posted with the online version of this article as a data supplement at jbjs.org (http://links.lww.com/JBJSREV/B147). This content was not copyedited or verified by JBJS.
